# Translational Challenges and Prospective Solutions in the Implementation of Biomimetic Delivery Systems

**DOI:** 10.3390/pharmaceutics15112623

**Published:** 2023-11-14

**Authors:** Zhe Wang, Xinpei Wang, Wanting Xu, Yongxiao Li, Ruizhi Lai, Xiaohui Qiu, Xu Chen, Zhidong Chen, Bobin Mi, Meiying Wu, Junqing Wang

**Affiliations:** 1 Department of Pathology, The Eighth Affiliated Hospital, Sun Yat-sen University, Shenzhen 518033, China; wangzh379@mail.sysu.edu.cn (Z.W.); lairzh3@mail2.sysu.edu.cn (R.L.); 2 School of Pharmaceutical Sciences, Shenzhen Campus of Sun Yat-sen University, Shenzhen 518107, China; wangxp39@mail2.sysu.edu.cn (X.W.); xuwt27@mail2.sysu.edu.cn (W.X.); liyx356@mail2.sysu.edu.cn (Y.L.); qiuxh27@mail2.sysu.edu.cn (X.Q.); chenx589@mail2.sysu.edu.cn (X.C.); chenzhd9@mail2.sysu.edu.cn (Z.C.); 3 Department of Orthopaedics, Union Hospital, Tongji Medical College, Huazhong University of Science and Technology, Wuhan 430022, China; mibobin@hust.edu.cn; 4 Hubei Province Key Laboratory of Oral and Maxillofacial Development and Regeneration, Wuhan 430022, China

**Keywords:** biomimetic, bioinspired, nanodiscs, liposomes, virus-like particles, albumin, ferritin, polysaccharides, extracellular vesicles

## Abstract

Biomimetic delivery systems (BDSs), inspired by the intricate designs of biological systems, have emerged as a groundbreaking paradigm in nanomedicine, offering unparalleled advantages in therapeutic delivery. These systems, encompassing platforms such as liposomes, protein-based nanoparticles, extracellular vesicles, and polysaccharides, are lauded for their targeted delivery, minimized side effects, and enhanced therapeutic outcomes. However, the translation of BDSs from research settings to clinical applications is fraught with challenges, including reproducibility concerns, physiological stability, and rigorous efficacy and safety evaluations. Furthermore, the innovative nature of BDSs demands the reevaluation and evolution of existing regulatory and ethical frameworks. This review provides an overview of BDSs and delves into the multifaceted translational challenges and present emerging solutions, underscored by real-world case studies. Emphasizing the potential of BDSs to redefine healthcare, we advocate for sustained interdisciplinary collaboration and research. As our understanding of biological systems deepens, the future of BDSs in clinical translation appears promising, with a focus on personalized medicine and refined patient-specific delivery systems.

## 1. Introduction

**Figure 1:**
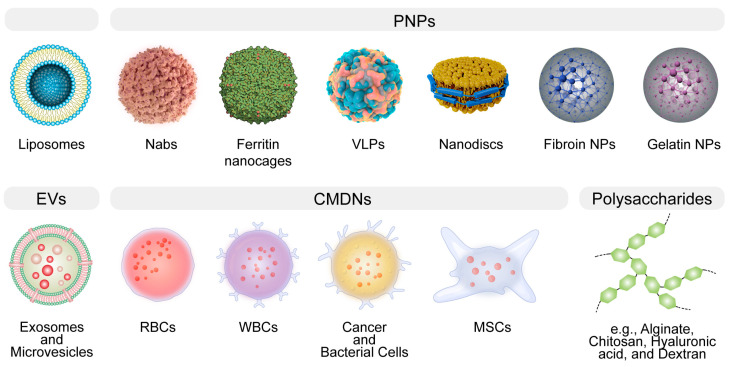
The general illustration of biomimetic delivery systems (BDSs). BDSs are designed to emulate natural structures, thereby augmenting therapeutic efficacy. Notable examples encompass liposomes, protein-based nanoparticles (PNPs), extracellular vesicles (EVs), cell membrane-derived nanocarriers (CMDNs), nanodiscs, and polysaccharides.

Biomimetic delivery systems (BDSs), defined by their ability to mimic biological systems, hold significant promise in the realm of biomedicine and nanomedicine. They leverage the principles of nature, emulating the structural or functional attributes of biological systems to enhance drug delivery capabilities [1,2,3]. BDSs often involve the use of naturally derived materials (Figure 1), the structural mimicry of biological entities, or the replication of biological processes, with the aim of improving drug delivery outcomes such as targeting ability, controlled release, and biocompatibility [4,5,6]. Recent advancements in biomimicry have resulted in the creation of innovative drug delivery systems [7,8,9] spanning various paradigms, such as liposomal carriers [10], virus-like nanoparticles (VLPs) for gene delivery [11,12,13], and hydrogel structures [14,15,16]. Additionally, new classes of delivery vehicles have emerged, including extracellular vesicles (EVs) [17,18], red blood cell (RBC)-based carriers [19,20], and nanodiscs (NDs), each presenting unique therapeutic prospects. EVs, naturally occurring cellular delivery systems, comprised of microvesicles and exosomes [21,22], hold promise due to their bio-compatibility and targeted delivery capability [23,24], stimulating interest in their use for delivering RNA-based therapeutics [21,25]. RBCs, with their advantageous properties such as a long circulatory half-life and immune evasion, are under investigation as potential drug carriers, with methods involving their engineering and manipulation into biomimetic nanoparticles [26,27,28]. NDs, mimicking high-density lipoproteins (HDL) [29,30], are versatile delivery platforms due to their ability to solubilize and present various drug molecules; additionally, they have potential benefits for targeted cancer therapy due to their preferential uptake by cancer cells [31,32].

The theoretical bedrock of biomimetic delivery systems (BDSs) is fundamentally rooted in the principles of self-assembly, molecular recognition, and biocompatibility [1,2,3]. Self-assembly refers to the process by which molecules spontaneously organize into ordered structures [33,34]. This characteristic, borrowed from nature, is widely harnessed to construct nanoscale delivery vehicles [35]. Molecular recognition refers to the ability of molecules to interact specifically with others, typically resulting in a biological function or response. This principle allows for the precise targeting of therapeutic agents to disease sites, minimizing off-target effects. Lastly, the nano-bio interface effect and biocompatibility are critical attributes of any biomimetic nanosystem intended for clinical use, ensuring that the system does not elicit adverse immune responses or toxic effects [36,37]. The paradigm of drug delivery has seen revolutionary advancement with the burgeoning interest in BDSs, which intimately mimic biological structures to enhance therapeutic efficacy [38,39,40].

These advancements have catalyzed previously unattainable therapeutic opportunities, including targeted cancer therapies [41], gene editing [42], and regenerative medicine [43]. The diversity and adaptability of these BDSs underscore the significant potential of leveraging nature’s design in the development of next-generation therapeutic interventions. However, the path from the bench to bedside translation is fraught with complexity. Despite the theoretical advantages of BDSs, their translation into clinical applications has been slower than expected, hindered by various technical, biological, and regulatory challenges. For instance, issues such as scalability of production, immunogenicity, stability of the systems under physiological conditions, and navigating regulatory approvals pose significant hurdles. The urgency for such a discourse is evident. The promise of biomimicry in healthcare can only be realized when these delivery systems transition from being experimental novelties to tools readily available in the clinician’s arsenal.

**Figure 2:**
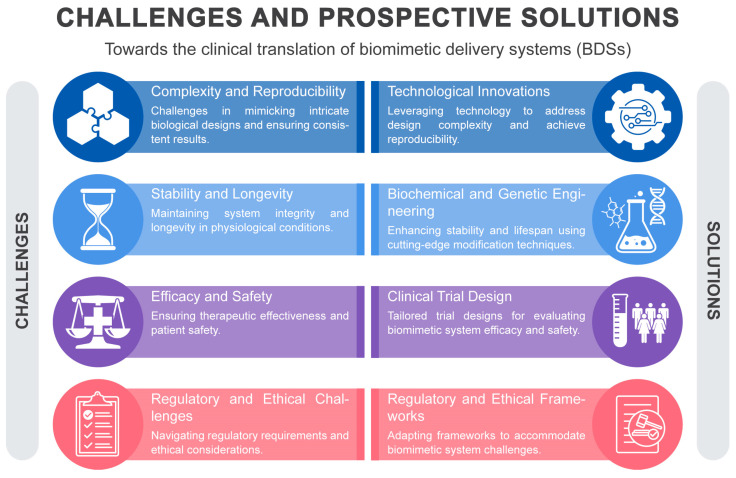
A schematic overview of the clinical translational hurdles and prospective solutions in BDS.

This review elucidates the translational challenges prevalent in the field, focusing on their intricate aspects and contemplating potential resolutions (Figure 2). Given the broad scope of this review, emphasis is placed on general themes rather than meticulous analyses of individual cases. We initially provide an overview of the strengths and weaknesses of various BDSs, then we examine challenges segmented into technical, biological, and regulatory categories before presenting emerging solutions and strategies, highlighted by instances of successful translation. Conclusively, we offer insights into the future challenges in the BDS field, emphasizing the revolutionary impact of these technologies on healthcare and advocating for sustained research and collaboration in this realm.

## 2. An Overview of the Strengths and Weaknesses of BDSs

In the rapidly evolving landscape of drug delivery, BDSs stand out as a beacon of innovation, drawing inspiration from biological structures and processes to optimize therapeutic delivery. By mimicking nature, BDSs aim to overcome the myriad challenges associated with traditional drug delivery, ranging from off-target effects to limited bioavailability [5]. BDSs span a broad spectrum, from liposomal structures to protein-based nanoparticles and CMDNs [1,8,42,44]. While the promise of BDSs is undeniable, it’s imperative to evaluate their strengths and weaknesses in comparison with each other (Table 1).

Liposomes are spherical vesicles composed of phospholipid bilayers that can encapsulate a wide variety of therapeutic agents. Their biocompatibility arises from their resemblance to biological membranes, making them a preferred choice for drug delivery [45]. Despite their adaptability in drug loading, liposomes are not without limitations [46]. A critical issue pertains to their stability, which can be compromised during storage, necessitating the development of sophisticated stabilization strategies to ensure the longevity and efficacy of the liposomal formulation [47,48,49,50]. In vivo, liposomes may exhibit rapid clearance from the bloodstream, primarily due to opsonization and subsequent phagocytosis by the cells of the mononuclear phagocyte system [51,52]. This necessitates careful consideration of liposome size, surface charge, and surface modification with polymers such as polyethylene glycol (PEG) to extend their circulatory half-life [51,53].

Protein-based NPs, encompassing albumin NPs, protein-based nanocages, VLPs, and NDs, offer a versatile toolkit for enhancing drug delivery, each with distinct advantages and shared challenges. Albumin NPs utilize human serum albumin, which has a natural propensity to bind to various substances, thereby facilitating the transport of a wide range of molecules [54]. The biodegradability and lack of immunogenicity of albumin contribute to its appeal as a drug carrier. Notably, albumin has a unique ability to accumulate in tumor tissues due to the enhanced permeability and retention (EPR) effect, making it particularly useful for oncological applications [55,56]. However, the drug loading efficiency of albumin NPs can be unpredictable, and their interaction with the biological environment may sometimes lead to rapid clearance from the circulatory system. Despite this, the clinical success of albumin NPs is exemplified by the FDA-approved drug Abraxane, which is an albumin-bound form of paclitaxel used for the treatment of various cancers [57]. Protein-based nanocages are a novel form of protein NPs that offer a highly structured and uniform platform for drug delivery [58]. They are engineered by utilizing the self-assembling properties of certain proteins to form cage-like structures that can encapsulate therapeutic agents within their hollow interior [59]. This allows for precise control over the dosage and protection of the cargo from enzymatic degradation. However, the complexity of synthesizing these nanocages poses a significant challenge, potentially limiting their rapid deployment in clinical settings [60]. VLPs are multiprotein structures that mimic the organization and conformation of viruses but are devoid of viral genetic material, which mitigates safety concerns associated with live viral vectors. The repetitive, high-density display of antigens on their surface makes VLPs particularly effective as vaccine platforms, eliciting strong immune responses [11,61,62]. However, the production of VLPs is technically demanding, often requiring cell culture systems, and the scale-up for mass production can be challenging [63,64,65]. Nanodiscs are synthetic nanoscale particles that incorporate membrane proteins within a phospholipid bilayer stabilized by scaffold proteins. NDs provide a unique milieu for the study of membrane proteins in their near-native state, which is invaluable for drug discovery and development [66]. While they offer a controlled environment for membrane proteins, their therapeutic application as drug delivery vehicles is still nascent [30,67,68], with issues related to production scalability and drug loading capacity yet to be fully addressed. In a comparative context, while albumin NPs have achieved clinical use, protein-based nanocages and VLPs are still primarily in the research or early clinical trial stages. NDs, being relatively recent developments, and have not yet been extensively explored for therapeutic delivery but hold potential due to their unique ability to present membrane proteins and delivery of lipophilic drugs. Each of these protein-based NPs has its advantages in terms of specificity, biocompatibility, and targeting ability; however, they also face common challenges such as production complexity, stability, and potential immunogenicity.

Silk fibroin (SF) and gelatin (GA) epitomize the contrasting paradigms within BDSs, each with inherent strengths and challenges. SF is distinguished by its robust mechanical properties and sustained release potential, making it a quintessential candidate for structurally demanding applications such as in bone tissue engineering and targeted cancer therapies [69,70,71]. Nevertheless, its utility is occasionally circumscribed by intricate processing requirements and immunogenic concerns. Conversely, GA is celebrated for its facile chemical modifiability and hydrogel formation aptitude, characteristics that are pivotal for localized therapeutic delivery and tissue engineering scaffolds [72,73]. Yet, its application is sometimes compromised by inferior mechanical integrity, thermal instability, and the latent risk of pathogenic transmission [74]. The selection between SF and GA for DDSs is thus dictated by a nuanced balance between the therapeutic context and the material’s physicochemical congruity, with each material offering distinctive contributions to the diversifying landscape of biomimetic therapeutic delivery.

EVs and CMDNs represent two innovative approaches in the realm of biomimetic drug delivery, each leveraging the innate properties of cellular components. EVs, owing to their natural origin, can transport a wide variety of biomolecules and have the ability to cross biological barriers with a low risk of immune response, positioning them as promising vectors for regenerative medicine and targeted cancer therapies [18,75,76]. Nevertheless, isolating EVs with high purity remains a significant technical challenge [77,78,79]. CMDNs, on the other hand, utilize the unique attributes of cell membranes to cloak nanoparticles, enabling them to evade the immune system and increase delivery specificity [27,80,81]. This strategy has shown considerable promise in targeted drug delivery and immunotherapy, capitalizing on the natural homing abilities of cells. Both EVs and CMDNs still face substantial production complexities (EVs in terms of isolation and CMDNs with membrane extraction and nanoparticle integration).

Polysaccharides, a diverse group of biopolymers, including alginate, chitosan, hyaluronic acid, and dextran, play a pivotal role in the landscape of therapeutic delivery due to their inherent biocompatibility and tailored biodegradability [82,83]. Alginate, renowned for its gel-forming capabilities, is widely used in wound healing applications and as a matrix for cell encapsulation, benefiting from its gentle gelation conditions that preserve cell viability [84,85]. Chitosan, with its distinctive mucoadhesive properties and ability to open tight junctions [86], is exploited for enhanced mucosal delivery of drugs, offering improved bioavailability and prolonged retention at the site of administration. Hyaluronic acid, by virtue of its specific interaction with CD44 receptors [87], which are overexpressed in many cancer cells, has emerged as a targeted delivery vehicle, especially in the treatment of osteoarthritis, where it can provide both viscosupplementation and targeted relief [88]. Dextran, due to its excellent solubility and minimal toxicity, is employed in various drug delivery systems and as a plasma volume expander, with its iron-conjugated forms used to treat iron-deficiency anemia [89,90]. 

Despite these advantages, the application of polysaccharides is not devoid of challenges; their susceptibility to rapid degradation in vivo may limit their utility, and potential immunogenicity cannot be entirely discounted. Moreover, the batch-to-batch variability and the complexity of producing highly purified, well-characterized polysaccharides can impact the reproducibility and scalability of pharmaceutical products. Hence, while polysaccharides offer considerable benefits for drug delivery, their clinical application requires meticulous optimization to ensure efficacy, safety, and manufacturability.

## 3. Challenges and Approaches in Clinical Translation of BDSs

### 3.1. Complexity and Reproducibility

In the realm of biomimetic delivery systems, different biomimetic materials and structures have been explored for their potential advantages in the delivery of therapeutic agents. Each of these systems brings unique complexities and challenges in terms of their production and ensuring their reproducibility (Table 2), which is vital for their successful translation into clinical applications.

Liposomes, vesicular structures composed of lipid bilayers, are valuable carriers for various drugs, improving their pharmacokinetics, biodistribution, and therapeutic index, exemplified by clinically approved liposomal drugs such as Doxil^®^/Caelyx^®^ and AmBisome^®^ [91]. However, challenges in clinical translation include the heterogeneous nature of liposomes affecting consistency between batches, impacting drug delivery efficacy and therapeutic outcomes [92]. Size and lipid composition variations, stability concerns related to environmental factors, and deviations in morphology and drug release under inappropriate storage temperatures or extreme pH levels are notable issues [47,51,93,94,95]. To mitigate these, real-time monitoring, process analytical technologies (PAT), and techniques such as nuclear magnetic resonance (NMR) spectroscopy and liquid chromatography–mass spectrometry (LC–MS) are crucial to ensure formulation consistency and rectify deviations immediately [96,97,98]. For instance, PAT provides real-time data that enables the monitoring and control of the manufacturing process, ensuring quality and consistency in the production of BDSs. LC-MS, on the other hand, is indispensable for the precise analysis of complex biodistributions and pharmacokinetics in BDSs, which is critical for the optimization of therapeutic delivery. Challenges in liposomal drug manufacturing include the need for meticulous control over storage and handling, stringent quality control, and managing the transition from the laboratory to the industrial scale, all contributing to increased costs and complexity [48]. However, continuous manufacturing processes and advanced technologies, such as high-throughput screening and microfluidic systems, can enhance consistency and uniformity, ensuring precise formulation control for therapeutic outcomes [99]. In silico methods aid in designing stable liposomal systems [100,101]. While it’s improbable to eradicate all challenges in liposomal drug delivery systems (LDDS), integrating advanced technologies can alleviate them, ensuring efficient and consistent production of clinically effective LDDS. The integration of these technologies into formulation and production processes is crucial in addressing the challenges comprehensively.

The exploration of endogenous proteins such as albumin in drug delivery is growing due to their biocompatibility and enhanced pharmacokinetics. However, the translation of albumin-based carriers is intricate due to challenges in modification and resultant variability [102]. The complexity arises from albumin’s tendency to undergo conformational changes and the presence of a single free thiol group that is reactive under physiological conditions, complicating the controlled modification. Additionally, albumin’s multiple drug-binding sites pose a challenge for achieving specific drug-to-protein ratios [103,104,105]. Methods such as covalent linkage and encapsulation are used for drug attachment to albumin [106], requiring precision to maintain albumin’s integrity, and inconsistencies in these processes can lead to variations in drug loading and reproducibility [107]. While albumin is naturally benign, modifications can potentially induce immune reactions, impacting its biocompatibility, binding affinities, biodistribution, and pharmacokinetics, thereby posing a risk of undermining its inherent benefits [104,108]. Such modifications and variability in drug release kinetics can influence drug efficacy, plasma levels, and safety [109,110]. Utilizing high-resolution techniques and computational modeling can provide structural insights and predict interaction behaviors in biological settings, helping in refining drug loading and streamlining the design process [111,112,113,114]. Scaling from the lab to the industrial level can impact product quality and characteristics in albumin-based systems, and the complexity of albumin modification challenges the reproducibility [102,115]. Implementing microfluidic devices [116,117], utilizing standardized albumin sources such as rHSA [118], and employing automated synthesis platforms can enhance reproducibility by ensuring consistent reactions and minimizing variability and contamination [119]. The incorporation of sensors and analytical tools for real-time feedback and continuous monitoring of synthesis parameters further ensures product consistency [120,121,122]. The complexity and need for precise reproducibility in albumin-based delivery systems pose significant challenges, but technological advancements, from high-resolution analyses to automation, combined with strategic design, address these challenges [54,123], paving the way for broader clinical adoption.

Ferritin-based PNPs show promise for personalized medicine due to their encapsulation abilities but face translation challenges stemming from the complexity and reproducibility of assembly [124,125,126]. Notably, ferritin assembly is governed by both pH and ionic strength, which exert their influence through the modulation of amino acid ionization states and subunit interactivity, respectively [125,127,128,129]. Variations in pH alter the protonation state of amino acids at the subunit interfaces, consequently affecting their charge and dictating the electrostatic landscape critical for subunit alignment and stabilization. Ionic strength contributes to this regulation by screening these electrostatic charges; elevated ionic strength can shield repulsive interactions, thereby promoting assembly, whereas diminished ionic strength may not provide adequate shielding, potentially leading to disassembly [125,130]. This delicate balance of physicochemical conditions is essential for the proper biological functioning of ferritin, as it dictates both the structural integrity and iron-storage capacity of the complex. Therefore, precise control of pH and ionic strength is critical due to ferritin’s conformational plasticity, and deviations can lead to irregular nanoparticles affecting drug delivery and therapeutic outcomes [129]. Standard assembly/disassembly methods and advanced spectroscopic techniques are pivotal for maintaining conditions and understanding ferritin conformational transitions [131,132]. Modifications to optimize encapsulation can disrupt self-assembly and affect size, thus impacting pharmacokinetics and pharmacodynamics [59,133,134]. Standardized modification protocols, including directed evolution and genetic fusion, are crucial for maintaining consistency [135]. The inherent size variability of ferritin nanoparticles poses further challenges [136], necessitating advanced separation methods and size-exclusion techniques to ensure uniform therapeutic outcomes [137]. Real-time monitoring and advanced characterization techniques such as cryo-electron microscopy provide insights into structures, aiding in addressing polydispersity [138]. Integration of technology advancements such as molecular dynamics simulations offers perspectives on ferritin assembly behavior, aiding in addressing the polydispersity [128,139] for informed design. A comprehensive approach focusing on control and standardization can help overcome challenges and realize ferritin’s clinical potential in personalized medicine.

Virus-like particles (VLPs) use the infectious properties of viruses for therapeutic delivery, relying on complex recombinant DNA technology [13], and face inherent production variability. Advanced bioinformatics tools can refine the integration of foreign DNA [140,141], reducing genetic risks and enabling exact cellular condition control, assisted by modern bioreactors and real-time monitoring [65]. These innovations, along with high-throughput screening and synthetic biology, can mitigate biological system variability and genetic instability, promoting consistent VLP manufacturing [141,142,143]. However, purifying VLPs is complex due to their similarity to host proteins and size variation. Variations in purification methods can affect VLP yield and characteristics [144], possibly causing inconsistent therapeutic results. Nanotechnology and advanced filtration [145,146], coupled with real-time monitoring and cutting-edge spectroscopy [147,148,149], address these challenges by distinguishing VLPs from impurities and ensuring structural integrity. A deeper understanding of fundamental biological processes and targeted interventions, backed by advancements in technology and knowledge, are crucial for developing more efficient and reliable production strategies for VLP delivery systems.

Nanodiscs (NDs), stabilized by membrane scaffold proteins (MSPs), are discoidal structures apt for studying membrane proteins and delivering bioactive agents due to their biomimetic nature [29,30]. However, their clinical application is hindered by challenges in the complex, multi-step assembly process and reproducibility. The assembly involves the self-assembly of phospholipids and MSPs, and the correct protein-to-lipid ratio is crucial for ND integrity and function [150]. Factors such as lipid type, MSP variant, and assembly conditions necessitate optimization and significantly impact the assembly complexity and reproducibility [150,151], which are essential for complying with strict pharmaceutical regulations. Minor variations could alter ND properties, affecting their in vivo behavior and therapeutic efficacy, leading to batch variability and translational challenges. Microfluidic automation [152], real-time monitoring [153], and design strategies, such as molecular dynamics simulations [154,155,156] can address assembly complexity and enhance understanding of ND behavior. The scalability of ND production is pivotal, with continuous flow synthesis being a potential solution to maintain quality and meet regulatory demands for manufacturing consistency, as traditional batch processes introduce variability and are challenging to scale [157,158]. Efficient detergent-removal strategies and the exploration of biocompatible, biodegradable detergents are vital to mitigate toxicity concerns and simplify post-assembly purification [159,160,161]. In conclusion, overcoming the challenges in assembly complexity, reproducibility, and scalability is crucial to harness the full potential of NDs in innovative therapeutic delivery systems.

Silk fibroin (SF) and gelatin (GA) have been extensively researched for their potential in biomimetic delivery systems, owing to their biocompatibility and adjustable degradation rates, essential for in vivo nanoparticle application, especially in drug delivery [72,73,74,162]. However, translational challenges arise from their inherent complexity and the associated reproducibility issues in nanoparticle fabrication. For SF, clinical application is hindered by product heterogeneity arising from variability in silk sources and fibroin properties [163]. Advanced genetic engineering tools, such as CRISPR/Cas systems, and standardized fibroin extraction methods can help overcome such variability, ensuring consistent quality and properties essential for drug delivery [164,165,166]. Similarly, GA faces variability and reproducibility challenges due to differences in source animals and extraction methods [167,168,169]. High-throughput screening techniques and process standardization [170,171,172], including controlled crosslinking conditions and microfluidic platforms [171,172,173,174], are crucial for maintaining consistency in nanoparticle production. These enhancements, along with computational models predicting interactions between SF or GA and encapsulated drugs, contribute to achieving optimal and consistent biological performance [175,176,177]. Thus, standardized sourcing, purification, and fabrication procedures coupled with a comprehensive understanding of their impacts are imperative for the successful clinical translation of these biomimetic systems.

Extracellular vesicles (EVs) are notable for their potential in targeted therapeutic delivery and have gained prominence in biomedical research due to their capacity to transfer cellular information. However, their clinical transition is impeded by challenges related to their production, heterogeneity, scalability, and stability [178,179]. EVs, originating from cell cultures, play roles in cellular communication and waste management but exhibit considerable variability in size, content, and origin, complicating manufacturing and impacting therapeutic predictability and reproducibility [180]. Controlling this variability is crucial and can be achieved using single-vesicle analysis techniques, such as nanoscale flow cytometry, and potentially through synthetic biology approaches to ensure uniform EV production [178,181,182,183]. Scalability remains a significant challenge, with existing methods such as ultracentrifugation being inefficient and inducing structural alterations in vesicles [184]. The introduction of novel technologies such as bioreactors and microfluidic platforms has revolutionized EV production by optimizing cell conditions and enhancing yield and process efficiency [185,186,187,188]. The stability of EVs is paramount, with external factors impacting their functionality and safety. Advanced lyophilization, nano-encapsulation, and cryoprotectants have been employed to enhance EV shelf life, protect vesicle integrity, and prevent aggregation [189,190,191]. The application of artificial intelligence and machine learning can expedite and standardize EV analysis for quality control [192]. Despite their immense therapeutic potential, the realization of EVs necessitates advancements in their biology, production optimization, and rigorous quality control to address the prevailing challenges.

Cell membrane-derived nanocarriers (CMDNs), particularly from erythrocytes, present a promising frontier in targeted therapeutic delivery due to their biological stealth characteristics [20,26,27]. Nonetheless, the complexities in isolation, modification, and loading processes, coupled with the need for rigorous quality control and reproducibility, impede their clinical translation [193]. The isolation of CMDNs is intricate, involving donor cell selection, cell lysis, and the removal of cellular components, and each stage introduces potential variability, affecting product consistency [194]. Donor cell selection, influenced by age, health, and genetics, affects nanocarrier characteristics and performance. Implementations of microfluidic technologies, automation, and the utilization of ‘cell banks’ with optimal donor cells can standardize processes and diminish variability [152,195]. Additionally, post-isolation engineering of CMDNs for enhanced stability, circulation, and targeted delivery introduces further complexity. Controlled conditions and precision are requisite for consistent modifications across batches, facilitated by techniques such as atomic layer deposition and bio-orthogonal chemistries [196,197], with real-time monitoring ensuring uniformity [198,199]. Rigorous validation is vital for confirming drug loading and release profiles, crucial for therapeutic efficacy. The need for stringent quality control amid varied CMDN properties necessitates comprehensive quality control approaches. Techniques such as nanoparticle tracking analysis and dynamic light scattering are fundamental for characterizing CMDN parameters [200]. However, inherent biological variability and multifaceted production processes exacerbate the challenges in capturing CMDN diversity. Feedback-controlled systems, such as process analytical technology (PAT) [201], and computational models leveraging molecular dynamics and machine learning provide predictive insights into nanocarrier behavior and aid in optimizing production parameters [202,203]. Overcoming the production complexities, variability, and quality control challenges is pivotal for the clinical realization of CMDNs.

Polysaccharides such as alginate, chitosan, hyaluronic acid (HA), and dextran are prominent in nanoparticle synthesis due to their biocompatibility and safety [204]. However, their natural origins introduce variability in source, purification, and modification, yielding heterogeneity in nanoparticle properties which can impact the stability and reproducibility of delivery systems. The diverse sources, with variations in biological, chemical, and physical properties, influence polysaccharide properties, such as molecular weight and degree of deacetylation, thereby affecting nanoparticle attributes such as size, charge, stability, and, ultimately, therapeutic efficacy [205,206,207]. Modern extraction techniques and purification processes can mitigate batch variability, while sensor-based technologies and process adjustments aim to enhance consistency [208,209,210,211]. However, residual contaminants and modifications to polysaccharides amplify heterogeneity issues, impacting solubility, degradation, and drug loading. The employment of machine learning and artificial intelligence optimizes modification parameters, ensuring consistent processes and reduced product variability. The inherent variability in polysaccharide-based nanoparticles alters biological interactions and poses challenges in clinical translation, affecting pharmacokinetics, biodistribution, and therapeutic efficacy [212]. Advanced characterization methods and real-time monitoring technologies, such as PAT and digital twins of the production process, are crucial to control heterogeneity and enhance reproducibility [201,213]. The inherent complexity and reproducibility challenges of polysaccharides necessitate the development of standardized methods for extraction, purification, and modification, as well as advanced characterization techniques. Integrating technological advancements and innovative design strategies is pivotal for developing consistent and effective polysaccharide-based delivery systems, essential for bridging the gap from laboratory to clinical application.

### 3.2. Stability and Longevity

The quest for the stability and longevity of BDSs in physiological conditions is a complex journey marked by numerous challenges (Table 3). These systems, while crafted to mimic the natural biological environment, still encounter substantial difficulties in withstanding rapid clearance or degradation within the human body. This factor reduces their therapeutic window, undermining their effectiveness in achieving the desired clinical outcomes.

Liposomes are inherently unstable due to the susceptibility of phospholipids to oxidation and hydrolysis, affecting their structural integrity and function [214,215]. Oxidation leads to the formation of cytotoxic peroxidation by-products, posing substantial challenges to clinical applications [52]. Antioxidants such as vitamin E and ferulic acid can neutralize oxidative damage, and their uniform distribution is facilitated by advanced techniques such as high-pressure homogenization [49,216,217]. The incorporation of stable phospholipids such as sphingomyelin and cholesterol can further enhance membrane stability [218]. Conversely, hydrolysis disrupts liposomal structure and compromises the stability and the encapsulated agents’ efficacy in physiological environments [219,220,221,222]. Encapsulation with lipid-polymer conjugates such as PEG-PE and emerging techniques such as electrospinning can mitigate hydrolytic degradation [223,224]. Utilizing hydrolytically stable phospholipid analogs and designing liposomes with interdigitated lipid phases or incorporating ceramides can also bolster resistance to hydrolytic degradation [225]. Therefore, a profound understanding of phospholipid oxidation and hydrolysis is essential for developing stabilization strategies, which are crucial for liposomes’ successful clinical translation.

Albumin-based BDSs, revered for their biocompatibility and molecule-binding potential, encounter numerous challenges in clinical transition due to their interactions with various bodily substances, leading to aggregation and premature therapeutic release [109,110,226]. Such interactions risk sub-optimal outcomes and affect pharmacokinetics and efficacy as they are quickly cleared by the immune system. Furthermore, enzymatic actions in the body can jeopardize their structural integrity and result in variable drug levels and adverse events [102]. Storage and transport also present challenges, including denaturation, oxidation, and aggregation [111,227,228,229]. Generally, protein-based BDS are highly sensitive to environmental conditions such as temperature fluctuations and light exposure, which can significantly compromise their structural integrity and stability [228,230]. Elevated temperatures may cause denaturation and aggregation of the biomimetic components while exposure to light, particularly UV light, can initiate oxidative reactions and photoinduced damage that further destabilize the protein structure. These changes not only lead to altered pharmacokinetics and reduced drug-binding efficacy, but also raise safety concerns due to the potential for increased immunogenicity. Thus, maintaining controlled storage and transport conditions is critical to preserve the functionality of protein-based BDSs. Several strategies have been developed to mitigate these challenges, including nanoencapsulation and PEGylation to prevent premature interactions and extend circulation half-life [231,232,233]. Modifying nanoparticle size and shape, utilizing enzyme-inhibiting coatings, and employing cryopreservation and lyophilization address issues related to immune evasion, enzymatic degradation, and structural integrity [234,235]. Implementations of antioxidants, hydrogel encapsulation, and optimized buffer solutions offer protection against various stresses and maintain albumin structure [236,237]. Molecular imprinting and stimuli-responsive elements have also been utilized for improved drug loading and controlled delivery [238,239,240,241]. Hence, integrating these methodologies is pivotal in addressing the complications associated with albumin-based BDSs, enabling enhanced therapeutic delivery.

Protein-based nanocages, led by ferritin, are a breakthrough in theranostic devices. They promise innovative drug delivery systems based on biomimetic principles. However, the journey to clinical use presents challenges, including structural disruption in varying in vivo environments, which might trigger unintended drug release [129]. These nanocages also risk denaturation, aggregation, or deactivation under certain conditions, necessitating specialized storage solutions. While ferritin’s capability to traverse biological barriers is notable, controlling sustained drug release remains complex [127,242], with modifications for targeted delivery potentially introducing immunogenicity [243,244]. Ensuring uniformity in properties and drug potency during clinical manufacturing is imperative. Addressing these challenges demands a multidisciplinary approach, employing advancements in material science, innovative storage technologies, molecular engineering for precise drug release, advanced bioconjugation, computational simulations, high-resolution analytics, and machine learning for real-time monitoring [245,246]. This integrated methodology, combining the expertise of nanotechnologists, biologists, and pharmacologists, is crucial for unleashing the full potential of ferritin-based systems in targeted oncology.

VLPs are renowned for their precise control, defined structures, and adjustable immunogenicity, marking them as ideal candidates for targeted delivery platforms. However, their stability is compromised in demanding physiological environments due to factors such as pH fluctuations and the presence of proteases, causing potential premature therapeutic release and impacting targeting capabilities [140,247,248,249]. The inherent immunogenicity of VLPs, while advantageous for vaccines, poses a significant challenge for drug delivery, as it can provoke immune responses leading to rapid clearance and possible side effects [12]. Addressing these issues involves incorporating pH-responsive modifications and protease-resistant motifs to enhance stability [250,251,252,253], leveraging nanotechnology and surface modifications to augment targeting precision [254,255,256], and developing innovative strategies including “stealth” VLPs and biomimetic coatings to balance immunogenicity [257,258]. Such developments are pivotal in evolving VLPs into efficient, stable therapeutic delivery systems poised to yield enhanced clinical outcomes.

NDs serve as versatile drug delivery platforms but are hampered by challenges stemming from their amphiphilic lipid nature, causing instability in size, shape, and functional efficacy. Factors including temperature, pH, and ionic strength can induce lipid phase transitions and nanodisc aggregation, potentially causing premature drug release and reducing therapeutic efficacy [259,260]. The vulnerability of NDs to oxidation and enzymatic degradation poses significant concerns regarding their longevity, and interaction with serum proteins can further induce instability [261]. Additionally, the formation of a protein corona can lead to swift immune clearance and can elicit immune responses, thereby raising safety concerns [262]. Strategies to enhance ND stability include reinforcing the lipid layer, incorporating antioxidants, PEGylation, and developing stimuli-responsive NDs, all of which are crucial to maintaining ND biocompatibility and therapeutic potency [29,40,263,264]. The advancement in these strategies holds the potential to revolutionize ND-based drug delivery systems.

Fibroin and gelatin, due to their biocompatibility and biodegradability, are widely used in biomimetic delivery systems but face challenges related to stability and longevity under physiological conditions [265,266]. These proteins are susceptible to enzymatic degradation and pH variations, which affect their structural integrity and could lead to premature therapeutic release. Additionally, traditional sterilization methods can compromise their structural effectiveness for drug delivery. Several strategies are being developed to overcome these challenges. Chemical crosslinking and blending with synthetic polymers enhance resistance to degradation and improve mechanical properties [267,268,269,270]. Integration of bioinert nanoparticles and lyophilization offers stability and controlled drug release [271,272]. Innovations such as pH-responsive coatings [273,274], coacervation- and electrospinning-optimized encapsulation technologies [275,276,277,278], and novel fabrication and sterilization methods, including supercritical carbon dioxide-based NP formation methodologies and cold plasma sterilization [71,279], are being explored to maintain material integrity and safety. These advancements reinforce the significance of fibroin and gelatin in evolving biomedical applications.

EVs exhibit promising capabilities for targeted therapies due to their unique biological functionality but face substantial challenges in maintaining stability and longevity [190,280,281,282]. Physiological factors, along with difficulties in isolation, purification, and modification, can alter EV structure and hinder therapeutic delivery capabilities [77,78,79,282,283]. The unstable nature of EVs necessitates advancements in methodology to preserve functionality during storage, transport, and therapeutic loading, with issues such as sensitivity to freeze–thaw cycles and long-term storage further complicating their utilization [191,284]. Strategies such as encapsulation technologies [285,286], surface modifications [287], and advanced isolation methods are being developed to address these challenges [187,188,288]. Additionally, innovations in cryoprotectants, packaging, and transport solutions are being explored to enhance EV stability and integrity [235,289]. The advancement of these strategies, coupled with interdisciplinary collaboration, is pivotal for harnessing the therapeutic potential of EVs in modern medicine.

In biomimetic delivery, CMDNs, particularly those derived from red blood cells (RBCs), display significant stability and longevity challenges and can trigger immune responses leading to premature clearance due to alterations during the extraction and modification processes [290]. The mononuclear phagocyte system (MPS) recognizes altered RBC-derived nanocarriers, reducing their bloodstream longevity [291]. Solutions including surface camouflage (immune evasion through surface engineering with biocompatible polymers such as polyethylene glycol (PEG) or proteins that mimic the natural RBC surface), synthetic RBC mimetics, and the controlled release (response to pH, temperature, or particular biomolecules) of immunosuppressive agents are being explored to mitigate these challenges and prolong circulation [292,293]. Furthermore, preserving structural integrity and maintaining optimal stability and efficacy during storage and transport is crucial, with enhancements via nanoengineering, refined cryopreservation, lyophilization methods, and innovative preservatives being pivotal [294,295,296]. The development of CMDNs necessitates a multidisciplinary approach, combining biotechnology, material science, and pharmacology, to optimize the stability, longevity, and controlled-release kinetics of RBC-derived nanocarriers, heralding advancements in therapeutic delivery systems.

Polysaccharide-based carriers such as alginate, chitosan, hyaluronic acid, and dextran exhibit unique stability issues. Alginate and chitosan are notable for their biocompatibility and biodegradability but are susceptible to instability due to their hydrophilic nature, resulting in vulnerability to environmental factors such as pH and ionic strength [297,298]. Chemical modifications and protective coatings can address these vulnerabilities, improving their resilience. Hyaluronic acid faces stability issues due to susceptibility to enzymatic degradation by hyaluronidases, affecting its longevity and therapeutic effect [299]. The introduction of enzyme inhibitors or structural modifications can improve its resistance. Dextran, while soluble and biocompatible, is sensitive to microbial contamination, affecting its long-term stability [299]. Enhanced sterilization, incorporation of antimicrobial agents, and encapsulation techniques can mitigate this susceptibility. The formulation of these polysaccharides into nanoparticles or microspheres offers improved stability and controlled therapeutic release, symbolizing a promising development in creating robust delivery platforms [204,211,300]. Furthermore, the profound potential of polysaccharide-based BDSs is notably challenged by inherent stability issues. The integration of technological advancements, innovative design, chemical modifications, and protective strategies is crucial for realizing their full therapeutic capabilities, promoting the development of more resilient and efficient delivery platforms. Despite the revolutionary prospects of these delivery systems in drug delivery, stability and longevity challenges in physiological conditions, storage, and transport require continuous research, development, and optimization of fabrication and handling processes. This emphasizes the need for stabilizing agents and optimized procedures to enhance the clinical translatability of these promising systems.

### 3.3. Efficacy and Safety

The efficacy and safety of therapeutic agents, especially BDSs that emulate natural biological entities, are fundamental to their clinical utility. These BDSs are anticipated to provide efficacy comparable or superior to existing treatments with a satisfactory safety profile, but their clinical translation encounters substantial challenges such as unpredictable in vivo behavior, potential off-target effects, and unexpected immune responses [301]. Comprehensive evaluation, including preclinical and clinical studies of pharmacokinetics, pharmacodynamics, and immunogenicity, is pivotal to establish therapeutic validity.

Liposome-based BDSs, noted for their ability to encapsulate diverse agents, promise enhanced drug solubility and targeted delivery [91]. However, intrinsic challenges exist, impacting therapeutic efficacy and safety [302]. Variations in entrapment efficiency can result in sub-optimal drug concentrations, affecting therapeutic outcomes. Challenges with drug release kinetics, premature or delayed, can compromise drug effectiveness [303]. Rapid clearance and degradation in biological fluids and interactions with serum proteins, enzymes, or immune cells diminish drug bioavailability [301]. Inaccurate targeting and off-target interactions can necessitate higher doses, inducing potential side effects. Liposomal formulations, especially those modified with targeting ligands, may elicit immune responses, ranging from allergies to severe anaphylaxis [304,305], and certain liposomal components can exhibit toxicity. The variability in the enhanced permeability and retention (EPR) effect introduces an additional complexity [306]. Rapid drug release due to destabilization presents overdose risks [307]. Despite the potential of liposomal systems, these multifaceted concerns necessitate meticulous consideration and ongoing refinement.

The clinical translation of liposomal technologies, exemplified by pioneering formulations such as Doxil^®^ and AmBisome^®^, highlights the innovation in therapeutic delivery. Doxil^®^, a paradigmatic FDA-approved nanodrug, utilized adaptive trial designs for dynamic dose adjustments, balancing efficacy with safety and showcasing the importance of real-time data-based refinements [308]. AmBisome^®^ distinguished itself with a meticulous comparative approach in clinical trials, revealing its superior therapeutic index in antifungal treatments [309,310]. The imperative theme is the necessity of adaptable and flexible trial designs; MAMS, or multi-arm multi-stage designs are a novel approach in clinical trial methodology, allowing for multiple treatments to be tested simultaneously against a common control group [311,312]. This design provides flexibility to add or drop treatment arms based on interim results. Therefore, MAMS designs are efficient by allowing simultaneous evaluations of various formulations, accelerating development and optimizing resource allocation. In the post-approval phase, the integration of real-world evidence (RWE) and stringent post-marketing surveillance are crucial, providing insights into long-term safety, rare side effects, adherence patterns, and therapeutic outcomes in diverse populations [313,314]. This approach, drawing from the foundational successes of Doxil^®^ and AmBisome^®^, informs and refines subsequent clinical trials and therapeutic guidelines. The clinical success of liposomal technologies underscores the essential role of innovative trial designs, adaptability, and ongoing evaluation in advancing liposomal therapeutics from experimental to established clinical treatments.

PNPs, encompassing a diverse set of biomaterials such as Albumin nanoparticles, protein-based nanocages (exemplified by ferritin), VLPs, NDs, fibroin, and gelatin, are advancing to the forefront of drug delivery research due to their inherent biocompatibility, biodegradability, and potential for precision-targeted therapeutic delivery. For Albumin nanoparticles, despite being synthesized from endogenous proteins, the inherent risk lies in the potential elicitation of immunogenic reactions, stemming from slight alterations or impurities during the nanoparticle formation process [315]; moreover, their inherent stability is also a concern, as degradation can substantially affect drug release kinetics, leading to suboptimal therapeutic effects [102,229]. Turning to protein-based nanocages, specifically ferritin, they display the dual challenges of potentially inconsistent drug loading efficiencies, which directly impact the therapeutic dosing [127,316], and a heightened sensitivity to environmental factors such as pH or temperature; this sensitivity might result in unintended, premature drug release [127,129]. Additionally, their natural role in iron storage poses concerns over inadvertently disrupting iron homeostasis in the body [317]. VLPs, while ingeniously designed to lack viral genetic material, are not without concerns, primarily rooted in the potential of evoking systemic immune reactions. Their complex synthesis pathway also introduces the risk of production inconsistencies and, albeit rarely, a shadow of concern regarding potential mutations, raising the specter of inadvertently reintroducing pathogenic properties [12,318]. NDs, in their design, carry lipid-based structures, which render them susceptible to oxidation or hydrolysis, challenges further exacerbated by potential size inconsistencies that can lead to variable biodistribution, affecting their therapeutic reach and efficacy [150]. Finally, the naturally-derived PNPs, fibroin and gelatin, introduce their own set of challenges: their natural sourcing can lead to variability in nanoparticle properties between batches, potential toxicity stemming from the use of chemical crosslinkers, and the concern of rapid degradation in physiological settings, which can obstruct the controlled, sustained release of therapeutic agents [162,167,173,319]. In summation, while the promise of PNPs in revolutionizing therapeutic delivery is undeniable, their path is fraught with multifaceted scientific challenges that mandate rigorous research and optimization before clinical fruition.

EVs and CMDNs, including exosomes, microvesicles, and apoptotic bodies, are prominent for their therapeutic delivery potential due to their biocompatibility and capability for targeted delivery, offering advantages over synthetic carriers. Nevertheless, integrating them into clinical paradigms requires rigorous evaluation of therapeutic efficacy and safety [320,321]. Achieving site-specific delivery is challenging, potentially leading to off-target effects [322]. Stability during storage is vital, with factors such as temperature fluctuations compromising therapeutic potential [190]. Immunogenicity is a significant concern; while autologous sources mitigate risks, large-scale production from allogenic or xenogenic sources amplifies associated risks [323]. Batch-to-batch variability and contamination risks during isolation compound safety concerns [324]. The potential for horizontal gene transfer by exosomes could inadvertently transfer detrimental genes. As the biomedical field progresses with the rise of EVs and CMDNs, there’s an escalating need for reconfigured clinical trial frameworks to address the unique challenges associated with these therapies, particularly due to variable cargo loading efficiencies influenced by variations in vesicular dimensions, intricate lipidomic architectures, and membrane biomechanics [325,326]. Adaptive clinical trial designs become indispensable, allowing for modifiable responses based on interim findings and leveraging real-time pharmacokinetic feedback to optimize dosages [327,328]. The MAMS designs are noteworthy, enabling concurrent evaluations to optimize therapeutic precision [329]. Integration of real-world data is crucial to understand the longitudinal stability and efficacy in real clinical settings, balancing trial controls with patient variability. Safety evaluations should consider the diverse origins of EVs, employing basket and umbrella trial structures to assess immunogenicity risks across different patient cohorts [330]. Sequential multiple assignment randomized trial (SMART) designs, renowned for flexibility, are pivotal to counter variability and contamination threats, allowing treatment recalibrations based on evolving responses or risks [331,332]. The latent risk in exosomes mediating detrimental horizontal gene transfers demands meticulous dynamic surveillance mechanisms supported by Bayesian analytical paradigms. In conclusion, to realize the potential of EVs and cell membrane-based nanocarriers without compromising safety, clinical trial methodologies must evolve, incorporating innovative, adaptive, and rigorous designs.

Polysaccharide-based BDSs are renowned for their biocompatibility, biodegradability, and functional modification capacities, making them prominent in drug delivery research [83,204,211,300]. However, alginate exhibits challenges including burst release patterns and syneresis, impacting optimal drug concentrations and release kinetics and posing potential overdose concerns [333,334]. Contaminants in alginate can also provoke inflammatory responses. Chitosan’s solubility is pH-dependent, affecting its efficacy in diverse bodily microenvironments, and variations in its molecular weight distribution can lead to discrepancies in drug loading and release profiles [298]. Its biodegradation kinetics can leave residual fragments in vivo, raising safety concerns including rare allergic reactions. HA’s propensity for rapid enzymatic degradation limits its suitability for sustained drug delivery, and its purity is crucial if derived from animal sources to avoid immune responses or pathogen transmission [299]. Dextran, though versatile, presents challenges, with variable molecular weights affecting delivery profiles, and has rare instances of induced anaphylactic reactions associated with higher molecular weights [205,335].

A holistic assessment of polysaccharide-based BDSs necessitates a transition from traditional to more innovative, flexible clinical trial designs, with adaptive designs becoming pivotal for modifications including dose titrations based on interim analyses, addressing biomimetics’ unpredictability [308,327,328]. The efficient MAMS design allows simultaneous evaluations of diverse formulations, swiftly sidelining suboptimal candidates, while platform trials provide a dynamic scaffold for continuous comparison of polysaccharide derivatives [312,328,329]. The specificity of umbrella and basket trials is invaluable for discerning patient subpopulations benefiting from particular formulations, enhancing precision medicine [330]. Integration of RWE is crucial, offering insights into broader clinical scenarios and assessing the real-world effectiveness of polysaccharide-based BDSs [314]. Incorporating patient feedback in patient-centric trials facilitates comprehensive assessments of biocompatibility and efficacy [336]. However, the employment of such innovative designs involves complexities; they require sophisticated statistical methodologies, continuous monitoring, and transparent, ethical decision-making. In summary, the clinical validation of polysaccharide-based BDS systems is intrinsically linked to the strategic employment of these innovative, nuanced trial designs in therapeutic applications.

In conclusion, while each biomimetic delivery system carries unique opportunities, they all share common challenges in terms of their in vivo behavior, safety, and efficacy profiles. To enable their clinical translation, a comprehensive understanding of these challenges and the development of strategies to address them is crucial. This should include extensive preclinical and clinical evaluation of their pharmacokinetics, pharmacodynamics, and potential for inducing immunogenicity. The successful resolution of these challenges will unlock the therapeutic potential of these biomimetic delivery systems, improving patient outcomes across a range of diseases and conditions.

### 3.4. Regulatory and Ethical Challenges

The clinical implementation journey of BDSs encompasses intricate regulatory necessities and significant ethical considerations, often aligning with advanced therapy medicinal products (ATMPs) or nanomedicines, requiring specialized regulatory pathways [337,338]. The diverse forms of BDSs, such as liposomes, albumin, CMDNs, and various polysaccharides, necessitate the formulation of innovative regulatory guidelines and consistent dialogue between researchers and regulatory entities to navigate the clinical translation pathway. Ethical considerations become paramount, especially with human-derived biomimetic materials such as EVs or RBCs [339], necessitating thorough informed consent processes, strict privacy protection measures, and equitable access considerations encompassing production cost, pricing, and healthcare infrastructure disparities. While addressing technical challenges is crucial, ethical concerns require equal emphasis, requiring a multidimensional approach to harmonize scientific innovation, regulatory compliance, and ethical responsibility in the clinical translation of biomimetic delivery systems.

Liposomes require intricate characterization due to their diverse properties, raising regulatory and informed consent complexities [46], and their predisposition to degradation necessitates stabilization efforts. PNPs such as albumin and ferritin pose risks of adverse immune reactions, batch variability, and contamination, particularly from animal-derived proteins, which elevate ethical concerns and can limit acceptability among certain demographics [340]. VLPs, although non-pathogenic, invoke apprehension about potential immunogenic responses and necessitate elevated consent standards due to uncertainties surrounding their long-term effects [64]. NDs, being relatively novel, face challenges in standardization and harbor unresolved ethical considerations. EVs present hurdles in achieving reproducible isolation and purification protocols and pose potential risks in transmitting undesired biomolecules, emphasizing the need for transparency [77,78,79,323]. CMDNs face challenges in preserving native membrane characteristics while balancing potential immunogenic reactions, especially when sourced from human tissues. Polysaccharides bring forth challenges related to consistency and contamination [211], with their derivation methods potentially conflicting with the preferences or beliefs of certain patient groups, thus intensifying ethical dilemmas.

The emerging BDSs epitomize the integration of nature’s complex designs with human technological developments and have brought to the forefront an urgent necessity for advanced regulatory and ethical frameworks tailored to their nuances (Table 4). Historically, the edifice of regulatory standards has been anchored on principles of safety, efficacy, and quality, further buttressed by ethical cornerstones such as informed consent, equitability, and transparency. These tried-and-true paradigms, though effective for conventional therapeutics, grapple with the multifaceted challenges inherent to BDS. A hallmark feature of these systems is their biological variability and complexity, which, while promising targeted precision, complicates the path to achieving consistent reproducibility, i.e., a gold standard in therapeutic evaluations. This variability is compounded by BDSs’ novel and potentially multifactorial mechanisms of action, which can diverge significantly from traditional therapeutics and demand a deeper level of scrutiny. Further, owing to their intimate mimicry of biological systems, BDSs introduce the possibility of unprecedented interactions with native biological entities, necessitating rigorous preemptive assessment and monitoring. On the ethical front, the material source variability of BDSs introduces intricate layers of concerns, spanning from informed consent and potential exploitation to uncharted territories of long-term biocompatibility and unforeseen systemic effects. The sophistication and innovation underlying BDSs, while promising groundbreaking therapeutic solutions, might also inadvertently escalate production and distribution costs, thus catalyzing debates on equitable accessibility, especially in socioeconomically diverse settings. As the biomedical community stands at this crossroads, a forward-thinking regulatory strategy is of paramount importance. This strategy should champion adaptive oversight mechanisms, foster interdisciplinary dialogues, and advocate for harmonized global standards, ensuring that BDS innovations are not siloed but shared collaboratively. Concurrently, ethical protocols require a renaissance, one that broadens the boundaries of informed consent, deepens stakeholder participation, and relentlessly pursues transparency, ensuring that the transformative potential of BDSs is harmoniously balanced with societal, moral, and patient-centric imperatives. The dawn of biomimetic delivery systems demands a rethinking of our regulatory and ethical scaffolds. While the challenges are intricate, they present an opportunity: to shape a future where innovation flourishes within robust societal safeguards, ensuring that advancements in drug delivery truly serve humanity’s best interests.

## 4. Conclusions

BDSs have emerged as a transformative frontier in nanomedicine, promising unparalleled advantages in drug delivery and therapeutic modalities. These systems, rooted in the principles of self-assembly, molecular recognition, and biocompatibility, encompass a variety of platforms such as liposomes [91], PNPs [30,74,162,242,341,342], extracellular vesicles [17], and polysaccharides [300]. Their clinical applications have been praised for achievements in targeted delivery, reduced side effects, and improved therapeutic outcomes. However, the journey of these innovative delivery systems from the lab bench to the bedside is not without its hurdles. The inherent complexity of biomimetic designs poses challenges in ensuring reproducibility, a crucial factor in clinical applications. The physiological environment presents issues related to the stability and longevity of these delivery systems. Moreover, the efficacy and safety of these novel therapies, although promising, need rigorous evaluation. Beyond the technical challenges lie intricate regulatory mazes and ethical considerations that must be navigated to achieve successful clinical translation. To overcome these challenges, the scientific community has turned to various strategies. Technological innovations have been at the forefront, addressing issues of complexity and reproducibility. The exploration and integration of advanced biomaterials aim to bolster the stability and lifespan of biomimetic systems in physiological settings. Recognizing the unique properties and challenges of biomimetic delivery, there has been a push for innovative clinical trial designs that can more aptly evaluate their efficacy and safety. Furthermore, it’s evident that the traditional regulatory and ethical frameworks might fall short, necessitating the evolution of these frameworks in alignment with the innovative nature of biomimetic delivery systems. Real-world case studies provide tangible evidence of these challenges and, more importantly, shed light on successful strategies and interventions that have paved the way for clinical translation. These instances not only offer insights, but also inspire confidence in the potential of biomimetic delivery systems to revolutionize healthcare.

The future holds substantial promise for the clinical translation of BDSs as advancements in understanding biological systems continue to refine the design and capabilities of BDSs. Anticipated innovations, emerging from interdisciplinary collaborations among biologists, chemists, engineers, and clinicians, will likely be more refined, efficient, and personalized, aligning with individual patient profiles for optimized outcomes. The evolving familiarity of global regulatory bodies with BDSs anticipates the establishment of more streamlined guidelines, expediting clinical translation. Initial challenges and learnings in clinical translation will be instrumental in refining subsequent iterations of BDSs for enhanced clinical application. In essence, BDSs, merging nature’s design with human ingenuity, have immense potential in revolutionizing drug delivery, and despite existing challenges, the commitment of the scientific community and ongoing technological and regulatory advancements underline a future replete with potential.

## Figures and Tables

**Table 1:** An overview of strengths, weaknesses, and therapeutic applications of BDSs.

BDS	Strengths	Weaknesses	Therapeutic Applications
**Liposomes**	Biocompatible, versatile in drug loading	Limited stability, potential for rapid clearance	Anticancer and antifungal therapy
**Protein-** **b** **ased NPs**			
Albumin NPs	Natural origin, good safety profile	Variable drug loading efficiency	Anticancer drug delivery
Protein-based nanocages	Defined structure, biodegradable	Complex production	Enzyme replacement therapy, vaccine delivery
VLPs	High immunogenicity, targeted delivery	Production challenges	Vaccines, cancer immunotherapy
NDs	Membrane protein stabilization, defined size	Limited drug loading	Drug and vaccines delivery, drug discovery
Silk Fibroin	Biocompatible, high mechanical strength, thermal stability	Potential immunogenicity, variable degradation rates, processing challenges	Bone tissue engineering, wound healing, anticancer drug delivery
Gelatin	Biodegradability, ease of modification	Potential risk of disease transmission, temperature sensitivity.	Drug delivery, tissue engineering
**EVs**	Natural origin, low immunogenicity	Isolation purity challenges	Regenerative medicine, anticancer therapy
**CMDNs**	Mimics natural cells, targeted delivery	Complex production	Targeted drug delivery, immunotherapy
**Polysaccharides**			
Alginate	Biocompatible, gel-forming	Rapid degradation in vivo	Wound healing, drug delivery
Chitosan	Biocompatible, mucoadhesive	Limited solubility in neutral and alkaline pH	Wound healing, vaccine delivery
Hyaluronic acid	Biocompatible, natural targeting to CD44 receptors	Rapid degradation in vivo	Osteoarthritis treatment, drug delivery
Dextran	Soluble, biocompatible	Potential for hypersensitivity reactions	Iron-deficiency treatment, drug delivery

**Table 2:** A summary of the complexities, reproducibility challenges, and prospective solutions related to various BDSs.

BDS	Complexity and Reproducibility	Prospective Solutions
**Liposomes**	Diverse lipids induce variability. Sustained stability is challenging. Surface alterations cause variability. Scaling up adds variability.	Advanced lipid-mixing technologies. Freeze–thaw increases reproducibility. Advanced ligand conjugation methods. Automated production control.
**Protein-** **b** **ased NPs**		
Albumin NPs	Influenced by albumin source. Uniform size and shape are difficult to attain. Altered surface for specific targeting. Efficient drug encapsulation control.	High-pressure homogenization. Improved purification techniques. High-throughput screening. Microfluidics and computational modeling.
Protein-based nanocages	Ensuring consistent protein folding. Reproducible encapsulation. Stable surface chemistry. Efficient drug encapsulation control. Consistent drug release profiles.	Advanced bioengineering methods. Monitoring protein folding in real-time. New modification methods for stability. Innovative drug-loading for consistency. Smart release systems for specific triggers.
VLPs	Complexity in VLP assembly. Attaining purity and reproducibility. Heterogeneous surface modifications. Inconsistent therapeutic encapsulation in VLPs.	Advanced purification such as SEC. Genomic engineering for optimized production. Developed specific bioconjugation techniques. High-throughput techniques for optimal encapsulation.
NDs	Component multiplicity causes variability. Consistent size and shape. Adding functional groups increases complexity. Batch-to-batch variability	Synthesis and purification for uniformity. Advanced assembly techniques. Site-specific functionalization and modular design. Standardized protocols, real-time QC, and advanced characterization.
Silk Fibroin and Gelatin	Source variability affecting properties. Controlling degradation profile. Ensuring efficient encapsulation. Batch-to-batch variability due to natural sourcing. Sensitivity to processing conditions leading to variability.	Implement strict source control and purification processes. Crosslinking and site-specific functionalization. Develop recombinant alternatives. Standardizing protocols. Quality assurance measures. Process analytical technology (PAT).
**EVs**	Heterogeneity of EV populations. Differentiating EV subtypes is challenging. Possible contamination with proteins. Ensuring efficient encapsulation. Controlling release kinetics. Maintaining EV properties post-modification. Ensuring targeting specificity. EV source depends on donor cells.	Advanced centrifugation. High-resolution imaging and flow cytometry. Improved purification processes. Sonication or electroporation. Covalent and non-covalent linking. Bio-orthogonal chemistry. Molecular imprinting techniques. Standardized cell lines/biofactories.
**CMDNs**	Potential heterogeneity due to cell sources. Unpredictable biological interactions. Batch-to-batch differences. Enhanced nanocarrier functionality/specificity.	Improved cell culture techniques. Predictive molecular modeling and simulation. Controlled nanocarrier production via microfluidics. Surface engineering, genetic modifications, molecular tethering strategies.
**Polysaccharides**		
Alginate	Variability in alginate source/purity. Gelation process control. Encapsulation efficiency variability.	Advanced chromatography for purification. Microfluidics for consistent gel bead formation. Advanced sonication/emulsification.
Chitosan	Molecular weight influences properties. Degree of deacetylation influences properties. Replicating desired structures is challenging. Crosslinking variability affects stability. Uniform surface properties are challenging.	Advanced chromatographic techniques to standardize molecular weight. Spectroscopy for precise deacetylation. High-resolution microscopy and automated synthesis. Advanced controlled crosslinking techniques. Advanced surface characterization.
Hyaluronic acid	Variability in sources. Consistent molecular weight is crucial.	Microbial synthesis of HA for consistency. Real-time molecular weight monitoring.
Dextran	Variability in molecular weight distribution. Branching variation affects behavior. Functional group variation. Achieving consistent size/morphology is challenging.	Controlled polymerization methods. Detailed structure analysis via spectroscopy. Controlled enzymatic/chemical modifications. Microfluidics for controlled and reproducible nanosystem generation.

**Table 3:** An overview of the stability, longevity challenges, and prospective solutions related to various biomimetic delivery systems.

BDS	Stability and Longevity Challenges	Prospective Solutions
**Liposomes**	Sensitivity to oxidation and hydrolysis. Fusion/aggregation in serum. Rapid clearance from circulation.	Liposome coating (e.g., PEGylation). Incorporation of cholesterol. Antioxidant inclusion.
**Protein-Based NPs**		
Albumin nanoparticles	Instability in harsh environments (e.g., acidic pH). Enzymatic degradation.	Cross-linking of albumin molecules. Encapsulation with protective polymers. Surface modifications.
Protein-based nanocages	Structural disintegration at non-optimal conditions. Immune recognition and clearance.	Chemical surface modifications. Incorporation of stability-enhancing ligands. Fusion with other stable proteins.
VLPs	Potential immunogenicity. Stability issues due to dynamic protein structures.	Genetic modifications. Encapsulation within protective matrices. Surface modifications to reduce immunogenicity.
NDs	Sensitivity to physiologic conditions, leading to structural alteration. Potential immune recognition.	Use of stable lipids. Protective protein inclusion. Surface modification.
Fibroin and Gelatin	Sensitivity to temperature and pH. Enzymatic degradation in vivo.	Chemical cross-linking. Incorporation into composite materials. Coating with protective polymers.
**EVs**	Susceptibility to clearance mechanisms. Sensitivity to physiologic conditions leading to vesicle disruption.	Surface modifications. PEGylation. Encapsulation within biomaterials. Cryopreservation techniques.
**CMDNs**	Potential immunogenicity. Sensitivity to in vivo degradation mechanisms.	Immune camouflage techniques. Genetic modifications for enhanced stability. Surface modifications.
**Polysaccharides**		
Alginate	Rapid degradation in vivo. Instability in the presence of divalent cations.	Cross-linking with divalent cations. Incorporation into composite materials. Layer-by-layer assembly.
Chitosan	Solubility issues in neutral and basic pH. Rapid degradation in vivo.	Chemical modifications for solubility. Cross-linking. Layer-by-layer assembly.
Hyaluronic acid	Rapid enzymatic degradation in vivo. Instability under harsh conditions.	Derivatization and cross-linking. Hydrogel formulations. Composite materials incorporation.
Dextran	Sensitivity to oxidative conditions. Enzymatic degradation.	Cross-linking. Encapsulation within protective matrices. Blend with other stable polymers.

**Table 4:** Overall insights into regulatory and ethical challenges for BDS.

Categories	Insights
Regulatory Challenges	Biological variability and complexity Achieving consistent reproducibility Potential unprecedented interactions with biological entities
Regulatory Frameworks	Adaptive oversight mechanisms Interdisciplinary dialogues Harmonized global standards
Ethical Challenges	Sourcing material from sentient entities (humans/animals) Informed consent and potential exploitation Equitable accessibility in diverse settings
Ethical Frameworks	Broadened boundaries of informed consent Stakeholder participation Pursuit of transparency

## Data Availability

Data sharing is not applicable.
